# Logistic regression analysis of pregnancy outcome and EFI score of infertile patients with ovarian endometriosis cyst after staged oral administration of traditional Chinese medicine

**DOI:** 10.3389/fendo.2025.1624925

**Published:** 2025-07-04

**Authors:** Yali Bo, Shaochan Peng, Jiawen Su, Dunmin Ye

**Affiliations:** Gynecology Department II, the Third Affiliated Hospital of Guangzhou University of Chinese Medicine, Guangzhou, Guangdong, China

**Keywords:** ovarian endometriosis cyst, staged oral administration of traditional Chinese medicine, pregnancy outcome, EFI score, infertility risk factors

## Abstract

**Background:**

Ovarian endometriosis cysts are associated with infertility, and recent research increasingly focuses on improving pregnancy outcomes. Traditional Chinese medicine (TCM) has long been used for gynecological issues, yet the impact of staged TCM administration on pregnancy outcomes and Endometriosis Fertility Index (EFI) scores in infertile patients remains underexplored.

**Objective:**

This study aims to evaluate the effects of staged TCM administration on pregnancy outcomes and EFI scores in patients with ovarian endometriosis cysts.

**Method:**

A retrospective analysis of 150 infertile patients treated with staged TCM administration was conducted. Patients received Formula I before ovulation and Formula II post-ovulation, with the cohort split into pregnancy (n=91) and non-pregnancy groups (n=49). All patients underwent laparoscopic surgery prior to TCM treatment. Logistic regression and ROC analysis were used to assess pregnancy predictors.

**Results:**

Significant differences in age, infertility type, infertility duration, r-AFS stage, cyst diameter, and EFI score (P<0.05) were found. Age, secondary infertility, infertility duration (≥3 years), advanced r-AFS stage, cyst diameter (≥3cm), and EFI score (<7) were key risk factors. The EFI score’s AUC for predicting pregnancy was 0.731 (95%CI, 0.617-0.844), with 72.58% sensitivity and 75.83% specificity. No significant difference in follow-up duration was observed between the groups.

**Conclusion:**

Age, secondary infertility, infertility duration (≥ 3 years), r-AFS stage (stages III and IV), cyst diameter (≥ 3cm), and EFI score (< 7) were identified as risk factors for infertility in patients with ovarian endometriosis following a two-phase staged oral administration of traditional Chinese medicine. Among these factors, the EFI score can serve as a crucial evaluation index for postoperative pregnancy, assessing the likelihood of pregnancy and predicting patient prognosis.

## 1 Introduction

Endometriosis (EMs) is a complicated chronic illness that affects 10% of women in their reproductive years globally. It may cause dysmenorrhea, persistent pelvic discomfort, and infertility (1). Approximately 30% to 50% of patients with EMs experience infertility, with ovarian endometriosis being the primary pathological subtype responsible for infertility, comprising 17% to 44% of cases (2–4). While laparoscopic surgery can efficiently eradicate ovarian endometriotic cysts and preserve fertility, the procedure escalates the likelihood of impairing ovarian function, thereby leading to a diminished postoperative pregnancy rate (5). Therefore, finding more effective treatment methods has become the focus of clinical research.

Traditional Chinese medicine (TCM), as a vital component of traditional medicine, has recently demonstrated unique advantages in the treatment of endometriosis (EMs). In TCM, endometriosis can be classified under categories such as “Zhengjia” (masses) and “infertility,” primarily attributed to liver and kidney deficiency, qi and blood insufficiency, qi stagnation, and blood stasis obstructing the uterus, leading to infertility. Blood stasis is considered the primary pathogenesis of this disease, and the treatment principle focuses on tonifying the liver and kidneys, invigorating blood circulation, and resolving stasis. The phased oral administration of TCM, which helps regulate the overall body condition and improve the local environment of the uterus and ovaries, has gradually become a viable option for treating endometriosis. The staged oral administration of TCM, involving a two-phase approach with specific herbal formulas administered before and after ovulation, helps regulate the overall body condition and improve the local environment of the uterus and ovaries. This method has gradually become a viable option for treating endometriosis. Therefore, conducting in-depth research on the pregnancy outcomes of EMs-related infertility patients following phased TCM treatment is crucial for identifying the key factors influencing therapeutic efficacy.

The Endometriosis fertility index (EFI) serves as a comprehensive metric for assessing endometrial reproductive function, amalgamating various clinical parameters pertinent to fertility, including endometrial thickness and blood flow dynamics. By quantifying the EFI score, patients’ fertility can be systematically evaluated, furnishing a robust foundation for treatment (6). However, there is a paucity of exhaustive investigations regarding the application of the EFI score in prognosticating the outcomes of staged oral administration of traditional Chinese medicine in individuals with endometriosis-associated infertility. Thus, against this backdrop, our institution undertook this study with the aim of elucidating the determinants influencing pregnancy outcomes following staged oral administration of traditional Chinese medicine in individuals with endometriosis-associated infertility through Logistic regression analysis. Furthermore, the study seeks to ascertain the efficacy of the EFI score in predicting treatment outcomes, thereby furnishing a more scientific and objective underpinning for the treatment and prognostic evaluation of individuals with endometriosis-associated infertility, thereby bolstering clinical decision-making processes.

## 2 Methods

### 2.1 Research flow chart

**Figure 1:**
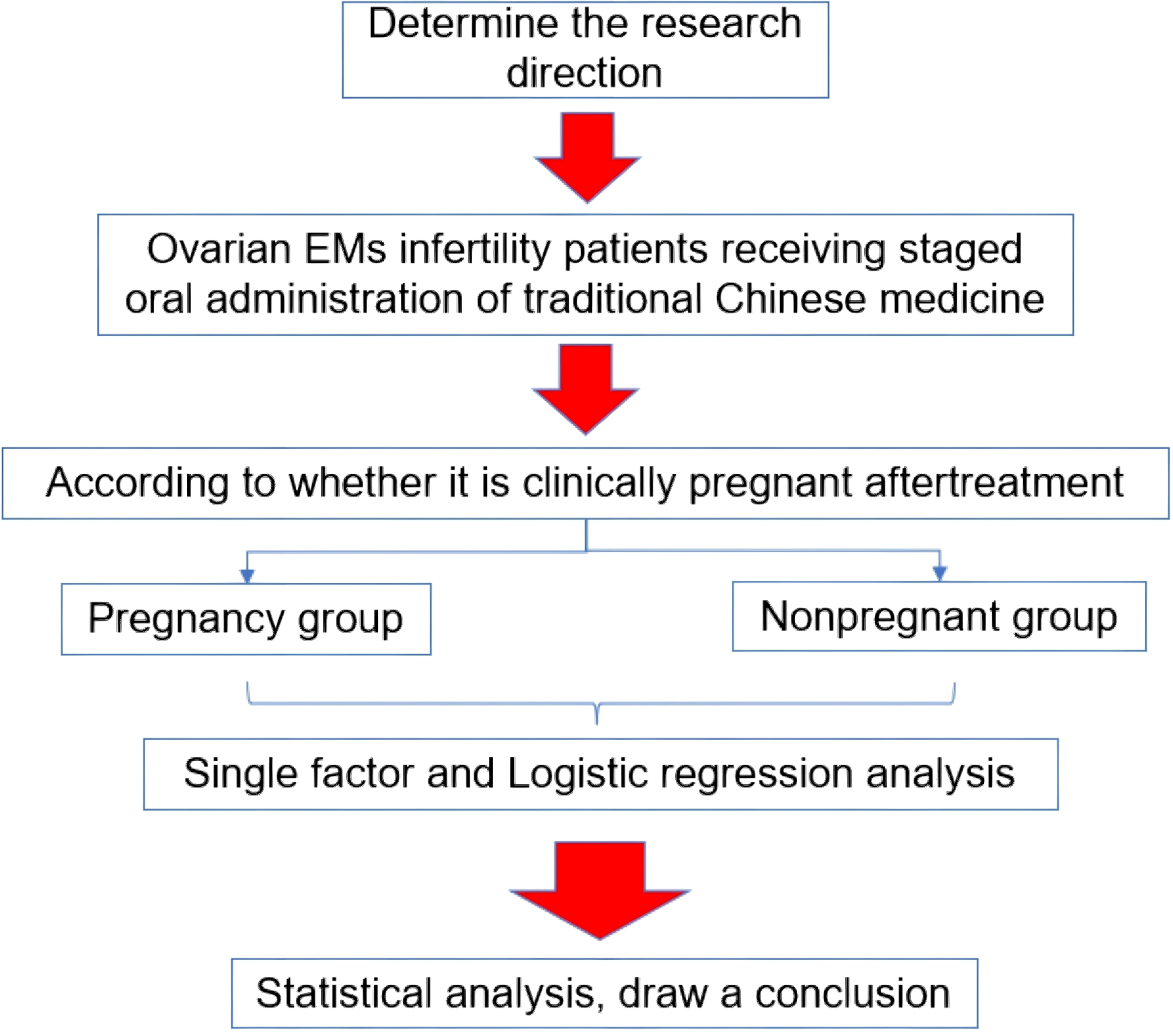
Flow chart of study design.

**Figure 1** shows the flow chart of this research.

### 2.2 Research object

Retrospective selection was used to gather clinical data from 150 infertile individuals with ovarian endometriosis who had treatment in our institution between November 2022 and November 2023. All patients underwent laparoscopic surgery to remove endometriotic cysts prior to initiating TCM treatment. The patients were between the ages of 25 and 43, with an average age of (31.15 ± 3.54). There were 54 instances of endometriosis in stage I, 45 cases in stage II, 27 cases in stage III, and 24 cases in stage IV, based on the revised American Fertility Society (r-AFS) classification. As for cyst types, there were 79 cases of single cysts and 71 cases of multiple cysts. Additionally, in terms of cyst location, there were 103 cases of unilateral cysts and 47 cases of bilateral cysts. The current study was approved by the Ethics Committee of the Third Affiliated Hospital of Guangzhou University of Chinese Medicine (approval number GCM202410189). Written informed consents from all patients were obtained in any experimental work with humans.

### 2.3 Entry and discharge standard

Inclusion criteria were as follows: (1) Patients were diagnosed with infertility at our hospital, following criteria outlined in relevant literature (7); (2) Ovarian endometriotic cysts were confirmed via pathology post-operation, using diagnostic criteria from pertinent literature (7); (3) All patients underwent staged oral administration of traditional Chinese medicine at our hospital.

Exclusion criteria were as follows: (1) Individuals with insufficient clinical information; (2) Patients with proximal tubal adhesion, uterine adhesion, and reproductive tract malformation; (3) Patients with recent abnormal semen examination; (4) Patients with other ovarian tumor diseases, recent history of surgery, and chemotherapy; (5) Patients with endocrine abnormalities such as thyroid dysfunction, hyperprolactinemia, and polycystic ovary syndrome; (6) Patients undergoing *in vitro* fertilization and embryo transfer.

### 2.4 Sample size calculation

Calculation formula of sample size:


$$n_{1}=\frac{[Z_{a/Z}\sqrt{p(1-p)(1+c)/c}+Z_{\beta }\sqrt{p_{1}(1-p_{1})+p_{2}(1-p_{2})/c{]}^{2}}}{{\left(p_{1}-p^{2}\right)}^{2}}$$

Bilateral α is 0.05, β is 0.20. This study is an experimental investigation, with the pregnancy rate of infertile patients with ovarian endometriosis cyst after staged oral administration of traditional Chinese medicine serving as the primary outcome measure. Referring to relevant literature, the estimated probabilities are denoted as follows: P1 = 0.95, P2 = 0.75. Upon calculation, the total sample size is determined to be 130 cases, factoring in a dropout rate of 15%, necessitating the inclusion of 150 patients.

### 2.5 Staged oral administration of traditional Chinese medicine

A two-phase staged oral administration of traditional Chinese medicine was implemented.

**TCM Decoction Method**: Add water to cover the herbs by 2 cm and soak for 30 minutes. Bring to a boil over high heat, then simmer over low heat for 30 minutes. The final decoction should be reduced to 200 ml. Take it twice daily, once in the morning and once in the evening, 30 minutes to 1 hour after meals, served warm.

**Ovulation Monitoring:** Starting on the 12th day of the menstrual cycle, use gynecological B-ultrasound to monitor follicle development. The formula No.1 was administered from the end of menstruation until ovulation. While the formula No.2 was administered after ovulation until the next menstruation or until pregnancy occurs, then stop.

Prescription I (Pre-Ovulation Phase): Administered from the end of menstruation until ovulation, this formula aimed to promote blood circulation and reduce stagnation. It comprised 10g Radix Notoginseng, 10g Radix Salviae Miltiorrhizae, 10g Rhizoma Chuanxiong, 10g Jiu Biying, 10g Lu Lutong, 15g Poria, 15g Rhizoma Dioscoreae, 10g Fructus Ligustri Lucidi, 20g Lycium chinensis, 15g Fructus Mori, and 6g Radix Glycyrrhizae.

Prescription II (Post-Ovulation Phase): Administered from ovulation until the next menstruation or until pregnancy was confirmed (at which point treatment ceased), this formula focused on nourishing qi and blood to support implantation. It included 20g Semen Cuscutae, 15g Herba Taxilli, 15g Radix Dipsaci, 10g Radix Salviae Miltiorrhizae, 15g Radix Codonopsis, 10g Rhizoma Atractylodis Macrocephalae, 15g Poria, 6g Radix Glycyrrhizae, and 10g Caulis Perillae.

Ovulation was monitored starting on the 12th day of the menstrual cycle using gynecological B-ultrasound to track follicle development, ensuring precise timing for transitioning between prescriptions. All herbal medicines were sourced uniformly from Daxiang Pharmaceutical Group Co., Ltd., through the hospital, ensuring consistent quality and composition. The staged administration was repeated each menstrual cycle over a six-month treatment period to evaluate therapeutic efficacy. Patients also received guidance on optimizing fertility and childbirth post-treatment.

### 2.6 Data collection and observation indicators

All data were retrieved from the electronic medical record system of the Digital Hospital Information Management Department of our institution. The collected data encompassed various parameters, including age, body mass index (BMI), type and duration of infertility, cyst diameter, mode of cyst dissection, cyst type (single or multiple), cyst location (unilateral or bilateral), presence of peritoneal endometriosis (PEM) and/or deep invasive endometriosis (DIE), severity of dysmenorrhea, revised American Fertility Society stage (rAFS), and postoperative Endometriosis Fertility Index (EFI) score. Additionally, the definition of “pregnancy” was standardized as the confirmation of a viable intrauterine pregnancy through ultrasound imaging. The EFI score, as per the scoring system detailed in literature (8), was calculated based on medical history factors: a score of 0 for age ≥ 40, 1 for ages 36-39, 2 for ages ≤ 35; 0 for primary infertility, 1 for secondary infertility; 0 for infertility duration > 3 years, 2 for ≤ 3 years. Moreover, information regarding whether patients underwent assisted reproductive technologies post-treatment was collected to account for potential confounding factors.

### 2.7 Statistical analysis

The data were analyzed by SPSS22.0 software. Counting data were presented as [n (%)] and assessed using the χ^2^ test. Measurement results with uniform variance and a normal distribution were written as ( ± s), and intergroup comparisons were conducted using independent sample t-test. Multivariate logistic regression analysis was used to explore statistically significant variables in univariate analysis. The Youden index was calculated for the EFI score to determine the optimal cutoff value. The receiver operating characteristic (ROC) curve analysis was used to assess the EFI score’s predictive ability for the outcome of pregnancy. All statistical tests were two-tailed, and a P-value<0.05 was considered statistically significant.

## 3 Results

### 3.1 Factors associated with pregnancy outcomes

According to whether the patients were pregnant or not after staged oral administration of traditional Chinese medicine, the results showed that there were statistical differences in the age, infertility type, infertility years, r-AFS staging, cyst dissection mode, cyst diameter and EFI score between the two groups (*P*<0.05). Additionally, there was no significant difference in follow-up duration between the pregnancy and non-pregnancy groups (P > 0.05). Moreover, a proportion of patients in both groups underwent assisted reproductive technologies; however, the difference between groups was not statistically significant (P > 0.05). Detailed outcomes are presented in **Table 1**.

**Table 1:** Pregnancy outcome after staged oral administration of traditional Chinese medicine in infertile patients with ovarian endometriosis cyst.

Grouping	Pregnancy group (n=91)	Non-pregnant group (n=59)	*t/χ2*	*P*
Age (years)	30.54 ± 4.81	35.06 ± 5.15	*5.467*	*<0.05*
BMI (kg/m^2^)			*4.288*	*>0.05*
<18.5	17 (18.68)	11 (18.64)		
18.5-24	68 (74.73)	38 (64.41)		
24-28	5 (5.49)	9 (15.25)		
>28	1 (1.10)	1 (1.69)		
Infertility type			*35.727*	*<0.05*
Primary	77 (84.62)	22 (37.29)		
Secondary	14 (15.38)	37 (62.71)		
Years of infertility (years)			*18.399*	*<0.05*
<3	70 (76.92)	25 (42.37)		
≥3	21 (23.08)	34 (57.63)		
Cyst location			*0.297*	*>0.05*
Unilateral	64 (70.33)	39 (66.10)		
Both sides	27 (29.67)	20 (33.90)		
Cyst type			*1.059*	*>0.05*
Single shot	51 (56.04)	28 (47.46)		
Multiple hair	40 (43.96)	31 (52.54)		
r-AFS Staging			*29.513*	*<0.05*
Stage I	44 (48.35)	10 (16.95)		
Stage II	31 (34.07)	14 (23.73)		
Stage III	9 (9.89)	18 (30.51)		
Stage IV	7 (7.69)	17 (28.81)		
Cyst exfoliation mode			*5.370*	*<0.05*
Clamp	81 (89.01)	44 (74.58)		
Physiological saline	10 (10.99)	15 (25.42)		
Diameter of cyst (cm)			*6.370*	*<0.05*
≥3	41 (45.05)	39 (66.10)		
<3	50 (54.95)	20 (33.90)		
EFI Scoring (points)			*30.118*	*<0.05*
<7	17 (18.68)	37 (62.71)		
≥7	74 (81.32)	22 (37.29)		
Degree of dysmenorrhea			*0.353*	*>0.05*
None	43 (47.25)	25 (42.37)		
Moderate pain	32 (35.16)	23 (38.98)		
Severe pain	16 (17.58)	11 (18.64)		
Merge PEM	19 (20.88)	14 (23.73)	0.610	*>0.05*
Merge DIE	43 (47.25)	34 (57.63)	1.542	*>0.05*

### 3.2 Logistic regression analysis of pregnancy predictors

**Figure 2:**
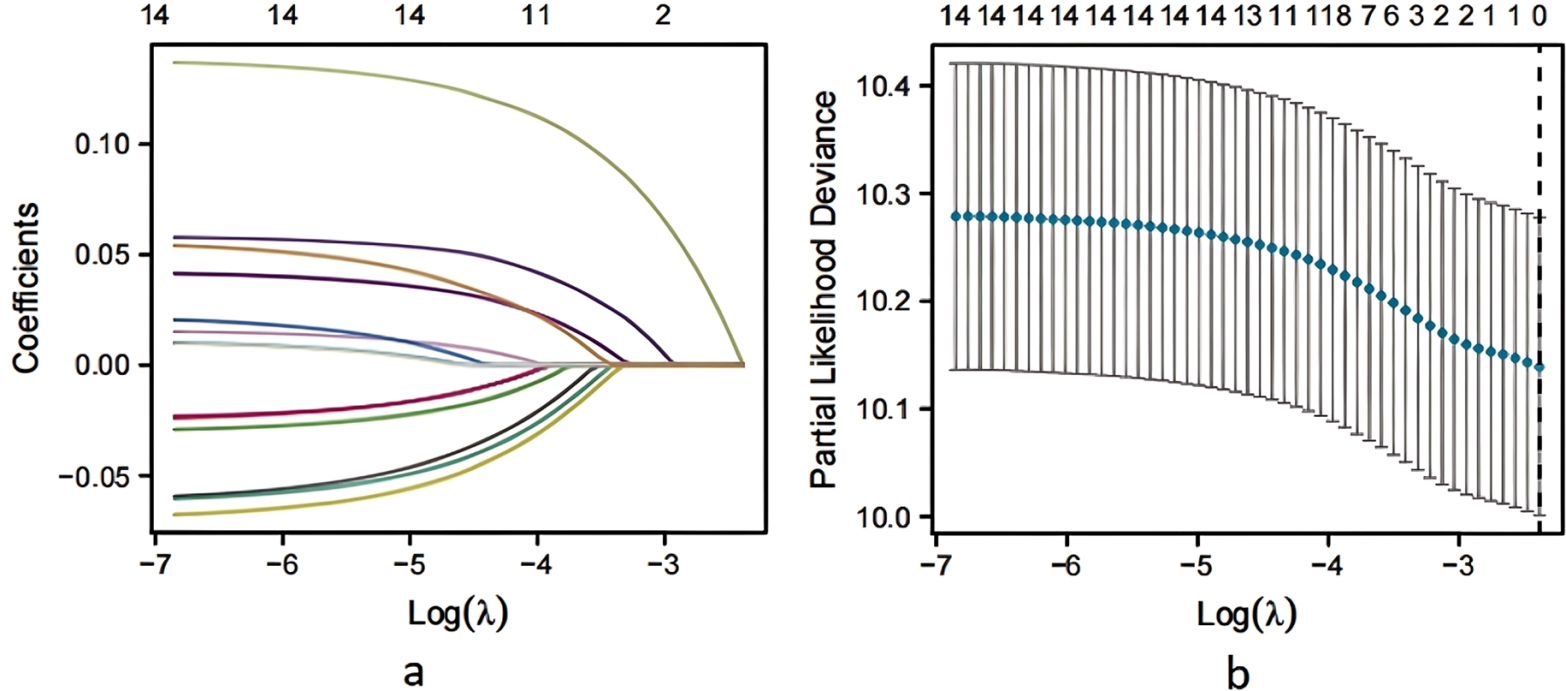
Screening of LASSO regression variables. **(a)** LASSO coefficient profiles of the variables plotted against log(λ). **(b)** Ten-fold cross-validation for tuning parameter selection in the LASSO model. Dotted vertical lines indicate the optimal log(λ) value corresponding to the minimum mean squared error.

Multivariate logistic regression analysis was conducted using the statistically significant factors identified in univariate analysis as independent variables and pregnancy outcome (yes = 1, no = 0) as the dependent variable. The assignment table detailing this analysis is presented in **Table 2**. The results of the analysis indicated that age, type of infertility (secondary), duration of infertility (≥3 years), r-AFS stage (predominantly stages III and IV), cyst diameter (≥3cm), and EFI score (< 7) were all associated with an increased risk of infertility in patients with ovarian endometriosis cyst following staged oral administration of traditional Chinese medicine (*P*< 0.05, **Table 3**). To identify key factors affecting pregnancy outcomes, we used lasso regression. **Figure 2a** shows how variable coefficients change with different λ values, and **Figure 2b** displays the partial likelihood deviance plot, helping determine the optimal λ for selecting predictive variables.

**Table 2:** Variable assignment table.

Related factors	Variable name	Variable assignment
Age	X_1_	Quantitative parameter
Infertility type	X_2_	Primary =0, Secondary =1
Years of infertility	X_3_	<3 years =0, ≥3 years =1
r-AFS Staging	X_4_	Stage I =0, Stage II =1, Stage III =2, Stage IV =3
Cyst exfoliation mode	X_5_	Clamp =0, Physiological saline =1
Diameter of cyst	X_6_	≥3cm=0,<3cm=1
EFI Score	X_7_	<7 Points =0, ≥7 Points =1

**Table 3:** Logistic regression analysis of multiple factors affecting the pregnancy outcome of infertile patients with ovarian endometriosis cyst after staged oral administration of traditional Chinese medicine.

Variables	β	S.E.	Waldx^2^	*P*	OR Value (95%CI)
Age	0.205	0.073	7.886	0.005	1.228 (1.064-1.416)
Type of infertility (secondary)	2.113	0.415	25.924	0.000	8.273 (3.668-18.660)
Years of infertility (≥ 3 years)	1.482	0.441	11.293	0.001	4.402 (1.855-10.447)
r-AFS Staging (High proportion of stage III and IV)	2.036	0.593	11.788	0.001	7.660 (2.396-24.490)
Cyst exfoliation mode	0.618	0.874	0.500	0.480	1.855 (0.335-10.289)
Diameter of cyst	0.789	0.328	5.786	0.016	2.201 (1.157-4.187)
EFI Score	1.915	0.437	19.203	0.000	6.787 (2.882-15.983)

### 3.3 The EFI score in predicting pregnancy outcome

**Figure 3:**
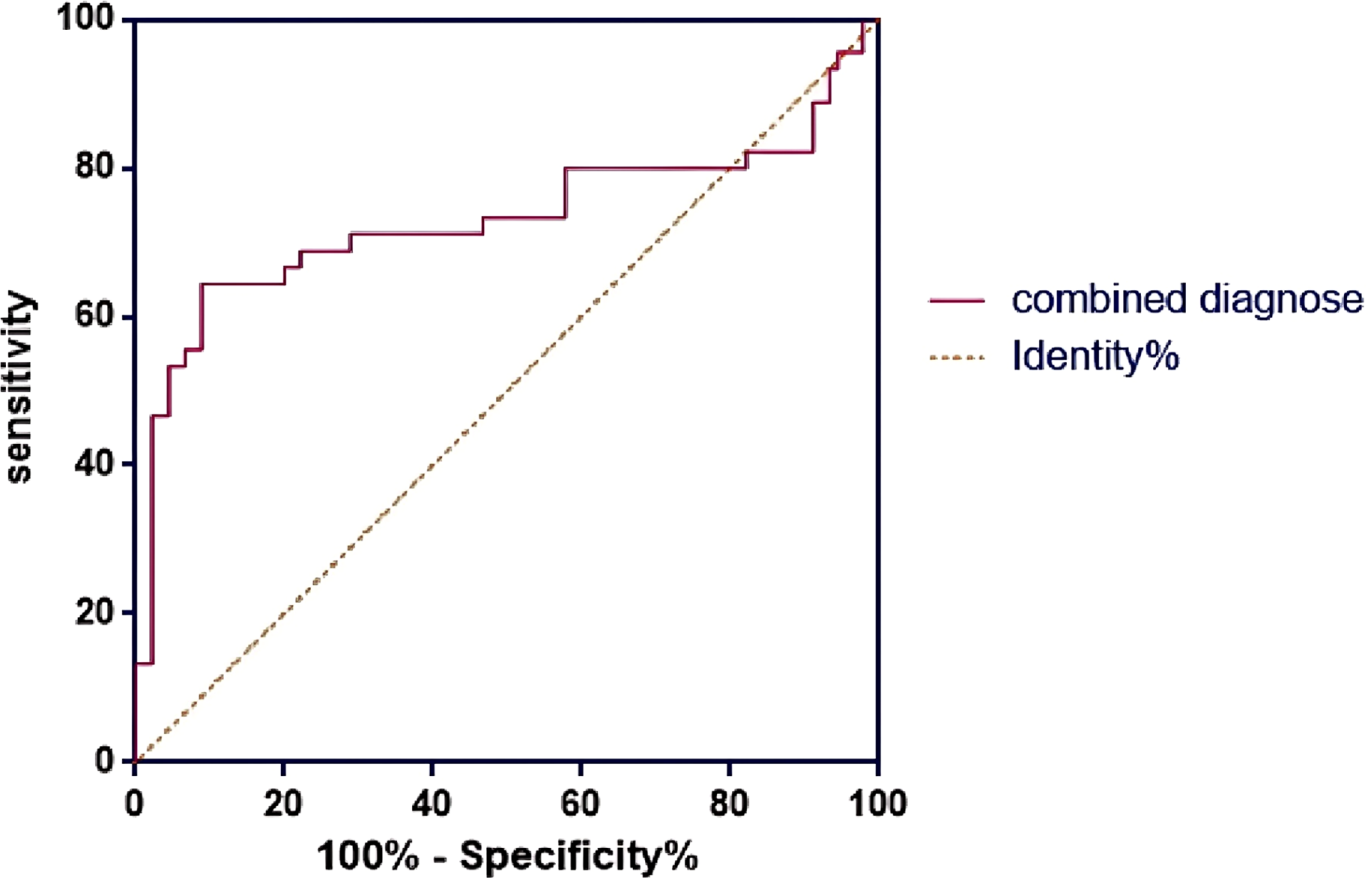
EFI score predicts ROC curve of pregnancy outcome.

The results of ROC curve analysis showed that the AUC of EFI score to predict the pregnancy outcome of infertile patients with ovarian endometriosis cyst after staged oral administration of traditional Chinese medicine was 0.731 (95%CI, 0.617-0.844), the sensitivity was 72.58%, and the specificity was 75.83% (**Figure 3**). The Youden index for the EFI score was calculated to be 0.484, suggesting an optimal cutoff point of 7 for maximizing the test’s effectiveness in distinguishing between patients who would achieve pregnancy and those who would not.

## 4 Discussion

The traditional Chinese medicine (TCM) used in this study is believed to enhance endometrial receptivity and ovarian function in patients with endometriosis (EM), while also promoting postoperative recovery and improving pregnancy outcomes. Modern pharmacological research indicates that Tanshinone in Salvia miltiorrhiza can inhibit the expression of VEGF and MMP-2, preventing the angiogenesis of ectopic endometrium and reducing blood supply to ectopic lesions, thereby inhibiting their growth (9). Ligustrazine, found in Ligusticum chuanxiong, can inhibit platelet aggregation and thrombosis, reducing fibrosis associated with EM by downregulating the expression of platelet-derived growth factor (PDGF) (10). Diosgenin in Dioscorea opposita regulates sex hormone levels, particularly estrogen receptors, and inhibits the excessive growth of the endometrium (11). Flavonoids in Taxillus chinensis can also reduce EM-associated fibrosis and improve liver and kidney function by inhibiting the TGF-β/Smad signaling pathway (12).

In this study, 91 patients with clinical pregnancy after staged oral administration of traditional Chinese medicine, the pregnancy rate was 60.67%, which was consistent with the previous report of 43%-64.8%. The study’s findings demonstrated that the age, type of infertility (secondary), years of infertility (≥ 3 years), r-AFS stage (high proportion of stage III and IV), cyst diameter (≥ 3cm) and EFI score (< 7) were all risk factors for infertility after staged oral administration of traditional Chinese medicine. Age is one of the key factors of pregnancy outcome in infertile patients with ovarian EMS after staged oral administration of traditional Chinese medicine (13). As women age, there’s a gradual decline in ovarian function, impacting both the quality and quantity of oocytes to varying degrees. Consequently, younger patients typically exhibit a higher likelihood of achieving pregnancy success (14). In addition, the type of infertility also has an important influence on the pregnancy outcome. Compared with patients with primary infertility, the situation of ovarian EMs complicated with infertility in patients with secondary infertility may be more complex, requiring more detailed staged oral administration of traditional Chinese medicine and individual intervention (15). Furthermore, the duration of infertility significantly influences pregnancy outcomes. Prolonged infertility may indicate sustained impairment of ovarian function, thereby limiting the efficacy of staged oral administration of traditional Chinese medicine (16). Hence, for patients experiencing long-term infertility, proactive treatment measures are warranted to enhance their chances of achieving pregnancy. In assessing ovarian EMs, while the correlation between r-AFS stage and postoperative pregnancy outcome may not be significant, the cyst diameter could indeed influence the likelihood of pregnancy success (17, 18). Larger cysts may exert greater compression and impair ovarian function, consequently diminishing the chances of successful pregnancy. Hence, during staged oral administration of traditional Chinese medicine, it is imperative to closely monitor cyst changes and promptly implement appropriate treatment measures.

The EFI score, serving as a pivotal tool for forecasting postoperative pregnancy prospects in infertile patients, was also validated in this study. Patients with elevated EFI scores generally exhibit more favorable pregnancy outcomes, whereas those with lower scores necessitate heightened focus on the restoration of ovarian function and the implementation of corresponding intervention measures (19, 20). However, the moderate predictive accuracy observed in this study underscores the necessity for integrating multiple prognostic indicators when evaluating patient prognosis. The EFI score of 7 serves as a pivotal threshold for clinical assessment, where fertility tends to be weaker when the score falls below this threshold. Furthermore, as the score diminishes, fertility is correspondingly compromised to a greater extent (4, 21). EFI score is an effective tool to evaluate the pregnancy prospect of patients with ovarian EMs complicated with infertility after staged oral administration of traditional Chinese medicine, and its diagnostic value has been further verified in this study. In this study, the analysis of the ROC curve revealed an AUC value of 0.731 for the EFI score, indicating a high level of prediction accuracy. Additionally, with sensitivity and specificity values of 72.58% and 75.83%, respectively, the EFI score demonstrates a degree of reliability and stability in predicting pregnancy outcomes. Based on the predicted results of EFI score, clinicians can evaluate patients’ pregnancy prospects more accurately and make more reasonable treatment plans. For patients with lower EFI score, the more attention should be paid to the recovery of ovarian function and strengthen staged oral administration of traditional Chinese medicine to improve the success rate of pregnancy (22, 23). Patients with higher EFI scores may undergo treatment with relative confidence and consider attempting pregnancy at an opportune moment (24).

This study has several limitations that merit acknowledgment. First, the retrospective nature of the analysis inherently limits causal inference and introduces the possibility of selection bias. Although rigorous inclusion and exclusion criteria were applied, and standardized laparoscopic and TCM treatment protocols were followed, unmeasured confounding factors may still influence the results. Second, the absence of a parallel control group prevents direct comparison with alternative treatment strategies or expectant management. While our intent was to evaluate outcomes in a homogeneously treated postoperative cohort, future randomized controlled trials are essential to validate these observations. Third, the single-center design may introduce selection bias and limit generalizability due to institution-specific practices; however, it allowed for highly standardized surgical and TCM treatment protocols. Nonetheless, the results should be interpreted with caution, as treatment outcomes may differ across diverse clinical settings and patient populations. Moreover, while the study identifies key clinical predictors of pregnancy outcomes, it does not explore the underlying biological mechanisms of TCM efficacy. Previous literature has indicated that several components of the administered formulas may influence inflammatory, hormonal, and immune pathways implicated in endometriosis (25, 26), and further molecular studies are needed to elucidate these effects. Finally, the follow-up duration was limited to short-term pregnancy monitoring. Longer-term outcomes, including live birth rates, recurrence of endometriosis, and sustained reproductive function, should be evaluated in future multi-center, prospective studies. Despite these limitations, the current study provides meaningful preliminary evidence for the potential role of staged TCM administration in improving postoperative fertility outcomes, and highlights the utility of the EFI score as a prognostic tool in this patient population.

## 5 Conclusion

In conclusion, the management of ovarian endometriosis-related infertility through a two-phase staged oral administration of traditional Chinese medicine requires careful consideration of multiple patient-specific factors. Age, type of infertility (secondary), duration of infertility (≥3 years), r-AFS stage (III and IV), cyst diameter (≥3 cm), and EFI score (<7) were identified as significant risk factors for successful pregnancy post-treatment. Utilizing the EFI score as a predictive tool enables clinicians to evaluate fertility prospects and tailor treatment strategies accordingly, thereby increasing the likelihood of pregnancy success in affected individuals. Future research should focus on expanding sample sizes and exploring the mechanisms underlying the efficacy of staged TCM treatments to further enhance clinical outcomes.

## Data Availability

The raw data supporting the conclusions of this article will be made available by the authors, without undue reservation.

## References

[1] Sindan N Bhandari A Sindan N Kc R Xia E Lin Y. Clinical factors influencing the pregnancy outcome after laparoscopic treatment in endometriosis-associated infertility patients: a retrospective study. Am J Transl Res. (2021) 13:2399–409. PMC8129327 34017398

[2] Vercellini P Vigano P Bandini V Buggio L Berlanda N Somigliana E. Association of endometriosis and adenomyosis with pregnancy and infertility. Fertil Steril. (2023) 119:727–40. doi: 10.1016/j.fertnstert.2023.03.018 36948440

[3] Taylor HS Kotlyar AM Flores VA. Endometriosis is a chronic systemic disease: clinical challenges and novel innovations. Lancet. (2021) 397:839–52. doi: 10.1016/S0140-6736(21)00389-5 33640070

[4] Shim JY Laufer MR. Adolescent endometriosis: an update. J Pediatr Adolesc Gynecol. (2020) 33:112–9. doi: 10.1016/j.jpag.2019.11.011 31812704

[5] Shim JY Laufer MR King CR Lee TTM Einarsson JI Tyson N. Evaluation and management of endometriosis in the adolescent. Obstet Gynecol. (2024) 143:44–51. doi: 10.1097/AOG.0000000000005448 37944153

[6] Gonzalez Salazar E Mastroianni G Vanetta C Goransky J Arbues G. Ileal endometriosis. An uncommon cause of bowel obstruction in women in fertile age. Medicina (B Aires). (2020) 80:566–9. 33048805

[7] T.C.M. Association. Clinical diagnosis and treatment guidelines. In: Obstetrics and gynecology division book. Beijing: People’s Health Publishing House (2007). doi: 10.3969/j.issn.1004-8189.2020.02.011

[8] Li T Zhai X. Predictive value of endometriosis fertility index for postoperative natural pregnancy in ectopic patients [in chinese]. Chin J Family Plann. (2020) 28:189–92. doi: 10.3969/j.issn.1004-8189.2020.02.011

[9] Chen ZZ Gong X. Tanshinone IIA contributes to the pathogenesis of endometriosis via renin angiotensin system by regulating the dorsal root ganglion axon sprouting. Life Sci. (2020) 240:117085. doi: 10.1016/j.lfs.2019.117085 31759042

[10] Huang S Xiao F Guo SW Zhang T. Tetramethylpyrazine retards the progression and fibrogenesis of endometriosis. Reprod Sci. (2022) 29:1170–87. doi: 10.1007/s43032-021-00813-x PMC8907108 35099777

[11] Yen ML Su JL Chien CL Tseng KW Yang CY Chen WF. Diosgenin induces hypoxia-inducible factor-1 activation and angiogenesis through estrogen receptor-related phosphatidylinositol 3-kinase/Akt and p38 mitogen-activated protein kinase pathways in osteoblasts. Mol Pharmacol. (2005) 68:1061–73. doi: 10.1124/mol.104.010082 15998873

[12] Qin M Huang Q Yang X Yu L Tang Y Zhang C. Taxillus chinensis (DC.) Danser: a comprehensive review on botany, traditional uses, phytochemistry, pharmacology, and toxicology. Chin Med. (2022) 17:136. doi: 10.1186/s13020-022-00694-5 36482376 PMC9730624

[13] Franca PRC Lontra ACP Fernandes PD. Endometriosis: A disease with few direct treatment options. Molecules. (2022) 27:4034. doi: 10.3390/molecules27134034 35807280 PMC9268675

[14] El Boghdady M Ewalds-Kvist BM. Laparoscopic surgery in patients with cystic fibrosis: A systematic review. Asian J Endosc Surg. (2021) 14:327–34. doi: 10.1111/ases.12874 33025750

[15] Shugaba A Lambert JE Bampouras TM Nuttall HE Gaffney CJ Subar DA. Should all minimal access surgery be robot-assisted? A systematic review into the musculoskeletal and cognitive demands of laparoscopic and robot-assisted laparoscopic surgery. J Gastrointest Surg. (2022) 26:1520–30. doi: 10.1007/s11605-022-05319-8 PMC9296389 35426034

[16] Connelly TM Clancy C Duraes LC Cheong JY Cengiz B Jia X. Laparoscopic surgery for complex Crohn’s disease: perioperative and long-term results from a propensity matched cohort. Int J Colorectal Dis. (2022) 37:1885–91. doi: 10.1007/s00384-022-04218-3 35869990

[17] Hudelist G Valentin L Saridogan E Condous G Malzoni M Roman H. What to choose and why to use - a critical review on the clinical relevance of rASRM, EFI and Enzian classifications of endometriosis. Facts Views Vis Obgyn. (2021) 13:331–8. doi: 10.52054/FVVO.13.4.041 PMC9148714 35026095

[18] Rodrigues DM de Avila I Amorim LVC Carneiro MM Ferreira MCF. Endometriosis fertility index predicts pregnancy in women operated on for moderate and severe symptomatic endometriosis. Women Health. (2022) 62:3–11. doi: 10.1080/03630242.2021.1986458 34852729

[19] Fruscalzo A Dayer A Londero AP Guani B Khomsi F Ayoubi JM. Endometriosis and infertility: prognostic value of enzian classification compared to rASRM and EFI score. J Pers Med. (2022) 12:1623. doi: 10.3390/jpm12101623 36294762 PMC9605607

[20] Tomassetti C. Why and when you should use the endometriosis fertility index (EFI). BJOG. (2020) 127:810. doi: 10.1111/1471-0528.16180 32105389

[21] Allaire C Bedaiwy MA Yong PJ. Diagnosis and management of endometriosis. CMAJ. (2023) 195:E363–71. doi: 10.1503/cmaj.220637 PMC10120420 36918177

[22] Amro B Ramirez Aristondo ME Alsuwaidi S Almaamari B Hakim Z Tahlak M. New understanding of diagnosis, treatment and prevention of endometriosis. Int J Environ Res Public Health. (2022) 19:6725. doi: 10.3390/ijerph19116725 35682310 PMC9180566

[23] Guidozzi F. Endometriosis-associated cancer. Climacteric. (2021) 24:587–92. doi: 10.1080/13697137.2021.1948994 34269136

[24] Pirtea P Vulliemoz N de Ziegler D Ayoubi JM. Infertility workup: identifying endometriosis. Fertil Steril. (2022) 118:29–33. doi: 10.1016/j.fertnstert.2022.03.015 35568524

[25] Wang T Guo R Zhou G Zhou X Kou Z Sui F. Traditional uses, botany, phytochemistry, pharmacology and toxicology of Panax notoginseng (Burk.). FH Chen: review. J ethnopharmacology. (2016) 188:234–58. doi: 10.1016/j.jep.2016.05.005 27154405

[26] Ding D Liu S Liu F Hao S Zhang C Shen Y. Exploring the role of Chinese herbal medicine in the long-term management of postoperative ovarian endometriotic cysts: a systematic review and meta-analysis. Front Pharmacol. (2024) 15:1376037. doi: 10.3389/fphar.2024.1376037 38910886 PMC11190181

